# Association of maternal BMI during early pregnancy with infant anemia: a large Chinese birth cohort

**DOI:** 10.1186/s12986-020-00448-w

**Published:** 2020-04-19

**Authors:** Shaohua Yin, Yubo Zhou, Hongtian Li, Zhihao Cheng, Yali Zhang, Le Zhang, Jufen Liu, Jianmeng Liu

**Affiliations:** 1 Institute of Reproductive and Child Health/National Health Commission Key Laboratory of Reproductive Health, Peking University Health Science Center, No. 38 Xueyuan Rd, Haidian District, Beijing, 100191 China; 2 Department of Epidemiology and Biostatistics, School of Public Health, Peking University Health Science Center, Beijing, China

**Keywords:** Infant anemia, Hemoglobin, Obesity, Chinese birth cohort, Prospective analysis

## Abstract

**Background:**

Infant anemia is prevalent in low- and middle-income countries. Maternal body mass index (BMI) is associated with serum ferritin in cord blood, but as yet has not been linked to infant anemia. The objective of this study was to examine the association of maternal BMI during early pregnancy with infant hemoglobin levels and anemia at 6 and 12 months in a Chinese birth cohort.

**Methods:**

The prospective cohort included 17,193 mother-infant pairs. Maternal weight and height prior to 20 gestational weeks as well as infant hemoglobin at 6 and 12 months were measured following standard procedures, and BMI was calculated as weight in kilograms divided by the square of height in meters. Women were categorized into underweight, normal weight (reference), overweight, and obesity. Infant anemia was defined as hemoglobin < 11.0 g/dl. Fractional polynomial regression was used to examine the relation between maternal BMI and infant hemoglobin, joinpoint regression to identify breakpoints, and logistic regression to estimate odds ratios (ORs).

**Results:**

In the cohort, 1160 (6.8%) were anemic at 6 months and 904 (5.3%) at 12 months. An inverse U-shaped relation of maternal BMI with infant hemoglobin was found at 6 months, at their maximum at maternal BMI of 22.4 kg/m^2^, and a similar relationship found again at 12 months. Maternal obesity rather than underweight was associated with an increased risk of anemia for infants at 6 months (adjusted OR 1.39, 95% CI 1.02, 1.88), but not at 12 months. Maternal anemia during mid-pregnancy augmented the risk at 6 months (adjusted OR 2.91, 95% CI 1.14, 7.46), but did not mediate the association (*Z* = − 1.102, *P* = 0.270).

**Conclusions:**

Maternal BMI during early pregnancy is correlated with infant hemoglobin in an inverse U-shaped profile, and obesity increases infant anemia risk that is aggravated by maternal anemia during pregnancy. This study enriched the epidemiological evidence on the adverse effect of high maternal BMI on long-term health of offspring. Optimizing maternal weight in obstetric care is necessary to improve offspring health.

## Introduction

The burden of obesity in women of reproductive age is increasing worldwide, particularly in low/middle-income countries. In China, about 14% of women of reproductive age were obese in 2008 [1], and likely worsening thereafter [2]. Maternal obesity increases the risk of various adverse pregnancy outcomes [3] and can affect offspring health [4, 5]. Previous studies showed that maternal obesity had been linked to maternal anemia [6, 7] and iron deficiency [8] that can predispose offspring to anemia [9, 10]; maternal obesity could reduce the iron store of newborn [11–14] that likely leads to anemia in later life [15]. However, direct assessment of the association between maternal obesity and offspring anemia is lacking.

On the other hand, underweight is also common in women of reproductive age in low/middle-income countries [16]. Maternal underweight has also been reported to increase maternal anemia during pregnancy [17, 18], likely predispose offspring to anemia. Again, no prior study has examined the association of maternal underweight with offspring anemia.

Comprehensive assessment of the association between maternal body mass index (BMI) and infant anemia is crucial for optimal periconceptional counseling regarding offspring health, especially given that as high as 273 million children under 5 years globally were afflicted with anemia [19], which can lead to irreversible health impairments [20, 21].

Therefore, we conducted this prospective cohort analysis to 1) describe the relationship between maternal BMI during early pregnancy and infant hemoglobin at 6 months and 12 months; 2) investigate whether maternal obesity or underweight is associated with infant anemia risk; 3) and assess whether the association (if exist) is mediated or modified by maternal anemia during mid-pregnancy.

## Subjects and methods

### Study subjects

**Fig. 1:**
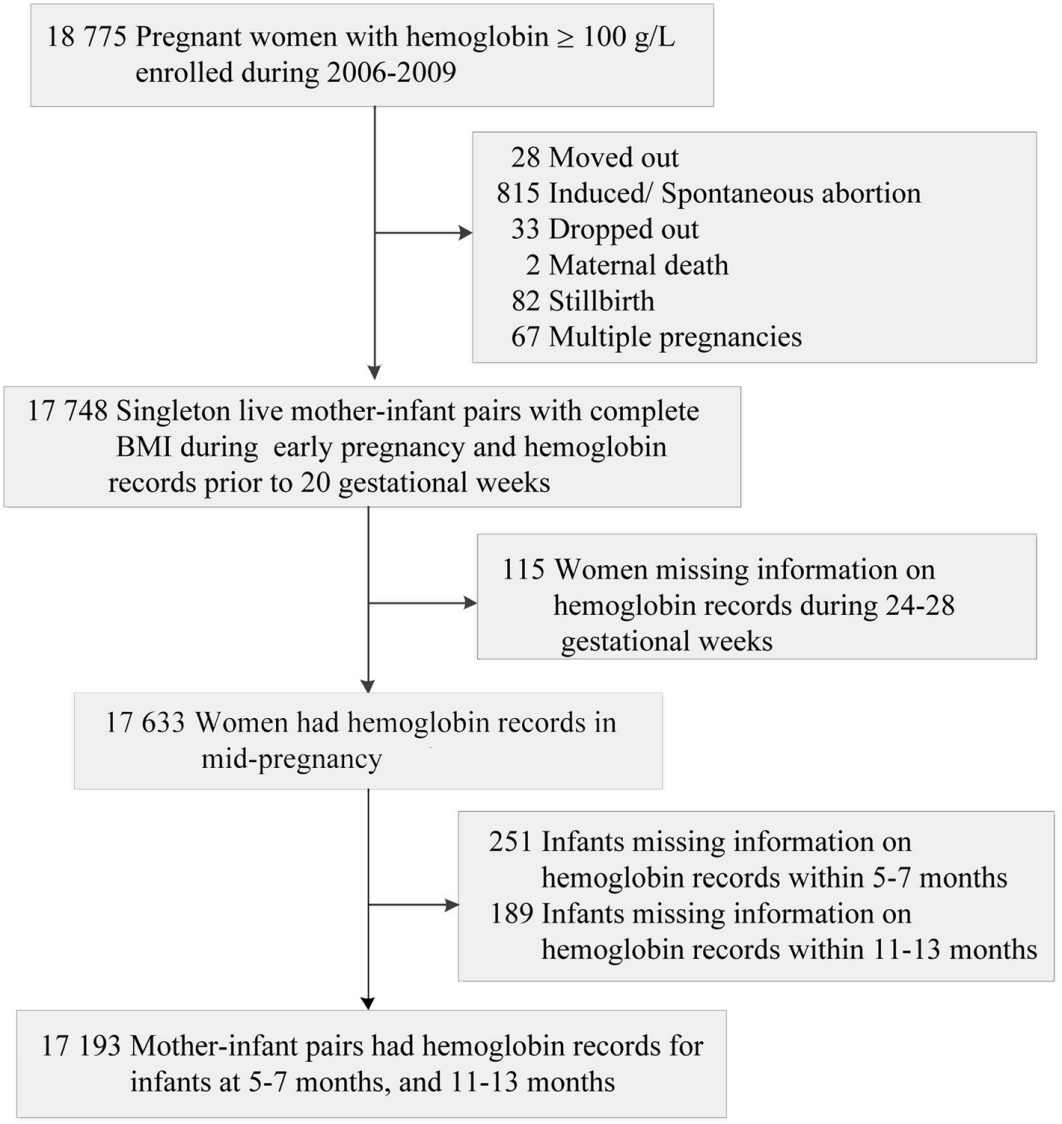
Flow chart of the mother-infant cohort

The cohort was drawn from a randomized controlled trial that was conducted in five counties in Hebei Province of northern China during 2006–2009, and all data were recorded in a perinatal and child health care surveillance system. Details of the trial have been described previously [22]. Briefly, nulliparous women were recruited from public clinics during their prenatal care; those with moderate or severe anemia (hemoglobin < 10.0 g/dl) were excluded. The 18,775 women were randomly assigned to receive daily folic acid, iron-folic acid, or multiple micronutrients from enrollment to delivery. For the present analysis, 1027 women were first excluded for moving out of the study region (*n* = 28), induced/ spontaneous abortion (*n* = 815), dropped out (*n* = 33), maternal death (*n* = 2), stillbirth (*n* = 82), or multiple pregnancies (*n* = 67). Among the remained 17,748 women who delivered single live births, 555 pregnant women were further excluded due to missing information on hemoglobin during mid-pregnancy (*n* = 115), missing their infants’ information on hemoglobin at 5–7 months (*n* = 251) or at 11–13 months (*n* = 189). Finally, 17,193 mother-infant pairs (91.6%) were left in the final analyses (Fig. 1). Maternal and infant characteristics between mother-infant pairs included (*n* = 17,193) and the excluded (*n* = 1582) did not differ, except for maternal age, occupation, maternal BMI, and infants’ sex, birth weight, and gestational age (*P* < 0.05). Maternal BMI was statistically comparable across supplement groups in the trial, and micronutrient supplementation was not related to maternal weight in the present study.

### Exposure and outcomes

Women’s weight and height wearing light indoor clothing and no shoes were measured by trained healthcare providers at enrollment before 20 weeks of gestation. Weight was measured to the nearest 50 g using an electronic scale (BW 150, UWE, Beijing, China) and height to the nearest 0.1 cm using a collapsible height board. The exposure in this study was BMI in early pregnancy of ≤12 gestational weeks. The early pregnancy BMI was calculated separately for women enrolled ≤12 gestational weeks and those enrolled during 13–20 gestational weeks. For women enrolled during 13–20 gestational weeks, weight in the first trimester was extrapolated using a standard formula: weight at enrollment – (gestational week at enrollment – 12 weeks) × 0.56 kg/week (the rate of gestational weight gain [GWG] in the second trimester for Asians) [23]. BMI during early pregnancy was calculated as first-trimester weight in kilograms divided by the square of height in meters. According to the World Health Organization (WHO) guidelines for Asians [24], maternal BMI was categorized as underweight, < 18.5 kg/m^2^; normal weight, 18.5–22.9 kg/m^2^; overweight, 23.0–27.4 kg/m^2^; and obesity, ≥ 27.5 kg/m^2^.

Maternal and infant hemoglobin levels were measured using a HemoCue photometric instrument (Model 201; HemoCue, Ängelholm, Sweden). Outcomes of interest in this study were infant hemoglobin levels measured at 5–7 months (mean: 6.3 months) and 11–13 months (mean: 12.3 months) and infant anemia which was defined as hemoglobin < 11.0 g/dl according to WHO recommendation [25].

Information on relevant covariates was also retrieved from the perinatal and child health care surveillance system.

### Statistical analysis

Categorical variables were presented as number (proportions), and continuous variables as mean (standard deviation, SD) or median (interquartile range, IQR). Chi-squared test was used to examine differences across maternal BMI groups for categorical variables, and analysis of variance or Kruskal-Wallis test for continuous variables.

Fractional polynomial model with generalized linear regression was used to examine the relation between maternal BMI and infant hemoglobin levels [26, 27], and joinpoint regression was applied to identify the potential breakpoints of the relation and to estimate respective regression coefficients. Logistic regression was used to estimate crude and adjusted odds ratios (ORs) of infant anemia across maternal BMI groups, with normal weight group as the reference. Adjusted covariates included maternal age (as a continuous variable), education status (primary school or less, middle school, and high school or above), ethnicity (Han or others), occupation (farmers or others), micronutrient supplementation (folic acid, iron-folic acid, and multiple micronutrients), anemia during mid-pregnancy (hemoglobin < 11.0 g/dl or not during pregnancy) [28], gestational week when hemoglobin was measured during pregnancy (as a continuous variable), rate of GWG in the second/third trimester (calculated by the difference between the last weight before delivery and the weight during early pregnancy and divided by gestational week, and then classified into quintiles within each BMI group), and infants’ sex (male or female), gestational age (< 37 or ≥ 37 weeks), mode of delivery (vaginal delivery, elective cesarean, and emergency cesarean), birth weight (< 2500, 2500–3999, and ≥ 4000 g), feeding mode (exclusively breast-feeding, most breast-feeding, partly breast-feeding, and formula feeding), and age of infant at hemoglobin measurement (as a continuous variable). Mediation and stratified analyses were further performed to examine whether maternal anemia during mid-pregnancy which was likely linked to offspring anemia [29], was a mediator or modifier for the association between maternal obesity and infant anemia.

To inspect the robustness of the results, the analyses regarding association of maternal obesity with offspring anemia were repeated either by using BMI cutoffs for western populations recommended by the WHO: underweight (< 18.5 kg/m^2^), normal weight (18.5–24.9 kg/m^2^), overweight (25.0–29.9 kg/m^2^), and obesity (≥ 30.0 kg/m^2^) [30], or by using the decile of maternal BMI as the classification criterion.

All analyses were performed using SPSS 24.0 (IBM Corp., Armonk. NY, USA), and a two-sided *P* < 0.05 was considered statistically significant.

## Results

Among the 17,193 mother-infant pairs, the median (IQR) maternal BMI during early pregnancy was 21.5 (20.0, 23.3) kg/m^2^, mean (SD) gestational age 38.9 (1.6) weeks, and mean birth weight 3297.6 (386.0) g; 16,988 (98.8%) were Han ethnicity and 15,644 (91.0%) farmers; 16,919 (98.4%) received a middle school education or above. Underweight, normal weight, overweight and obese mothers were 1507 (8.8%), 10,829 (63.0%), 4124 (24.0%), and 733 (4.2%), respectively. Infants born to obese mothers were more likely to be males, to have small gestational age and large birth weight, and to be born by cesarean delivery (Table 1).

**Table 1:** Maternal and infant characteristics according to maternal BMI during early pregnancy ^a^

Characteristic	Underweight (*n* = 1507)	Normal weight (*n* = 10,829)	Overweight (*n* = 4124)	Obesity (*n* = 733)	*P*
**Mother**					
Age (years)	23.1 ± 2.4	23.5 ± 2.6	24.0 ± 3.2	24.4 ± 3.7	< 0.001^*^
Weight (kg)	47.1 (45.0, 50.0)	55.0 (51.9, 57.55)	63.0 (60.1, 66.45)	76.3 (72.3, 81.2)	< 0.001^*^
Height (cm)	160.0 (158.0, 164.0)	160.0 (157.0, 163.0)	160.0 (157.0, 162.0)	160.0 (157.0, 163.0)	< 0.001^*^
Early pregnancy BMI (kg/m^2^)	17.8 (17.2, 18.2)	20.9 (19.9, 21.9)	24.2 (23.5, 25.3)	29.3 (28.3, 31.0)	< 0.001^*^
Rate of gestational weight gain (kg/week)	0.6 ± 0.2	0.5 ± 0.2	0.4 ± 0.2	0.4 ± 0.2	< 0.001^*^
Micronutrient supplementation					0.709
Folic acid	515 (34.2)	3610 (33.3)	1385 (33.5)	227 (31.0)	
Iron-folic acid	505 (33.5)	3613 (33.4)	1388 (33.7)	244 (33.3)	
Multiple micronutrients	487 (32.3)	3606 (33.3)	1351 (32.8)	262 (35.7)	
Education status					< 0.001^*^
Primary school or less	29 (1.9)	148 (1.4)	75 (1.8)	22 (3.0)	
Middle school	1202 (79.8)	8719 (80.5)	3268 (79.2)	617 (84.2)	
High school or above	276 (18.3)	1962 (18.1)	781 (19.0)	94 (12.8)	
Han ethnicity	1490 (98.9)	10,710 (98.9)	4067 (98.6)	721 (98.4)	0.341
Farmer occupation	1348 (89.5)	9864 (91.1)	3742 (90.7)	690 (94.1)	0.003^*^
Anemia during mid-pregnancy	119 (7.9)	670 (6.2)	256 (6.2)	37 (5.1)	0.033^*^
**Infant**					
Male sex	767 (50.9)	5712 (52.8)	2137 (51.8)	404 (55.2)	0.206
Birth weight (g)	3193.1 ± 353.4	3280.1 ± 368.7	3354.5 ± 407.6	3448.1 ± 474.9	< 0.001^*^
Gestational age (weeks)	39.0 ± 1.6	38.9 ± 1.6	38.8 ± 1.7	38.7 ± 1.8	< 0.001^*^
Mode of vaginal delivery	863 (57.3)	5921 (54.7)	1781 (43.2)	210 (28.7)	< 0.001^*^
Feeding mode					0.176
Exclusively breast-feeding	480 (31.9)	3440 (31.8)	1249 (30.3)	231 (31.5)	
Most breast-feeding	364 (24.1)	2471 (22.8)	1005 (24.4)	176 (24.0)	
Partly breast-feeding	549 (36.4)	4044 (37.3)	1522 (36.9)	251 (34.2)	
Formula feeding	89 (5.9)	667 (6.2)	263 (6.4)	51 (7.0)	
Missing	25 (1.7)	207 (1.9)	85 (2.0)	24 (3.3)	

^a^ Unadjusted means ± SDs or median (IQR) were computed for continuous variables, and number (proportions) for categorical variables

^*^P < 0.05 (Chi-squared test for categorical variables, and analysis of variance or Kruskal-Wallis test for continuous variables)

### Maternal BMI and infant hemoglobin

**Fig. 2:**
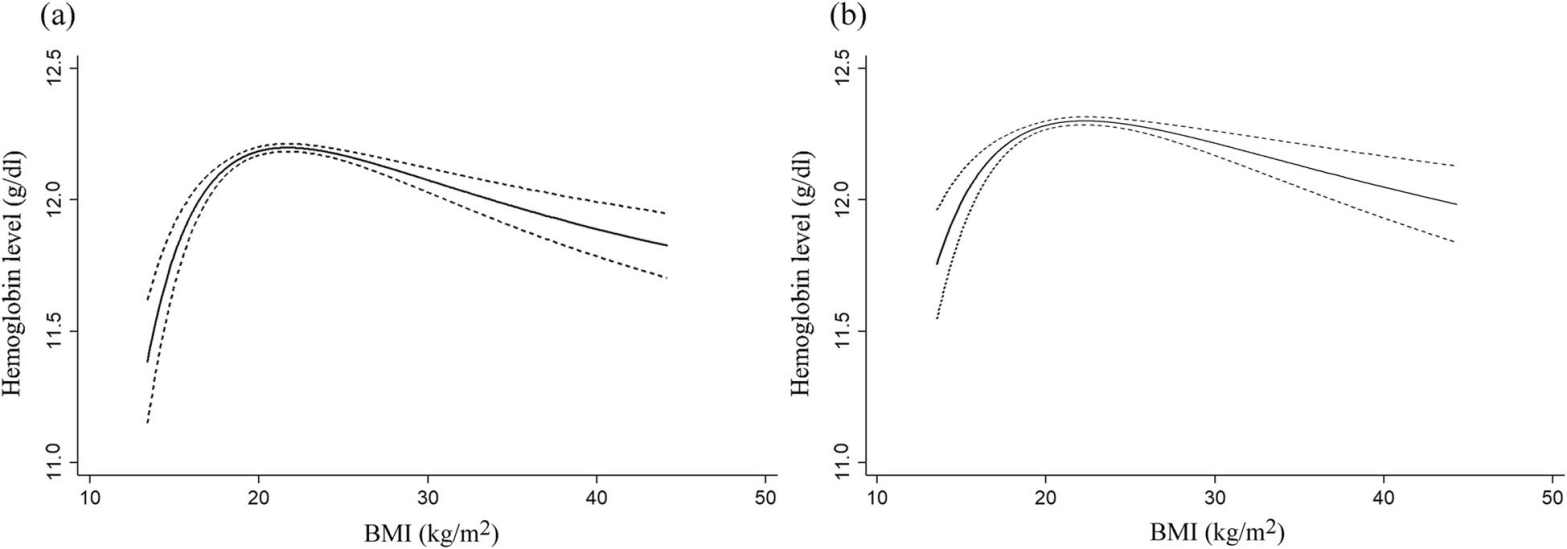
Relations of maternal BMI during early pregnancy with infant hemoglobin levels; hemoglobin levels, solid lines; 95% CIs, broken lines. **a** Relation of BMI during early pregnancy with hemoglobin for infants at 6 months. **b** Relation of BMI during early pregnancy with hemoglobin for infants at 12 months

The median (IQR) hemoglobin levels were 12.2 (11.6, 12.8) and 12.2 (11.7, 12.8) g/dl for infants at 6 months and 12 months, respectively. Fractional polynomial regression showed an inverse U-shaped relation between maternal BMI during early pregnancy and hemoglobin levels for infants both at 6 and 12 months (*P values* < 0.01 for nonlinearity, power *p* = − 2 and q = − 2), with an apex at 22.4 (95% CI 21.6, 23.2) kg/m^2^ of maternal BMI for 6-month old infants, and 22.6 (95% CI 21.6, 23.6) kg/m^2^ for 12-month old infants (Fig. 2). That is, hemoglobin levels tended to be lower in infants born to mothers with both low and high BMI, indicating a potential positive relation between maternal BMI and infant hemoglobin when BMI was less than 22.4 kg/m^2^, and a negative relation otherwise (Table 2).

**Table 2:** Regression coefficients (95% CI) estimated by segmented regression

Maternal BMI during early pregnancy	*β*^a^	SE	95% CI
Infant at 6 months			
BMI < 22.4 kg/m^2^	0.028^*^	0.006	0.017, 0.039
BMI ≥ 22.4 kg/m^2^	−0.024^*^	0.005	−0.033, −0.015
Infant at 12 months			
BMI < 22.6 kg/m^2^	0.021^*^	0.005	0.011, 0.032
BMI ≥ 22.6 kg/m^2^	−0.018^*^	0.004	−0.027, −0.010

^a^ Regression coefficient was estimated by joinpoint regression

^*^P < 0.01

### Maternal obesity and infant anemia

Overall prevalence of infant anemia was 6.8% (1160/17193) at 6 months and 5.3% (904/17193) at 12 months. In crude analyses, maternal obesity was associated with increased risk of infant anemia at 6 months (OR 1.41, 95% CI 1.08, 1.84), but not at 12 months (OR 0.94, 95% CI 0.66, 1.33). The ORs were not materially changed after controlling for covariates, regardless of whether GWG rate, mode of delivery, birth weight, and maternal anemia during mid-pregnancy were controlled or not (Table 3). Mediation analysis did not find that maternal anemia in mid-pregnancy mediate the association of maternal obesity with infant anemia at 6 months (Z = − 1.102, *P* = 0.270) (Additional file 1, Fig. S1). In analysis stratified by maternal anemia in mid-pregnancy, we observed no significant interaction between maternal anemia and BMI (*P*_-interaction_ = 0.130), while maternal anemia in mid-pregnancy appeared to augment the risk of infant anemia at 6 months (adjusted OR 2.91, 95% CI 1.14, 7.46) (Additional file 2: Table S1).

**Table 3:** Crude and adjusted ORs (95% CI) of infant anemia for maternal BMI during early pregnancy

Infant anemia	Early pregnancy BMI, *n* (%)			
	Underweight	Normal weight	Overweight	Obesity
Anemia at 6 months ^a^	107 (7.1)	688 (6.4)	301 (7.3)	64 (8.7)
Crude OR	1.13 (0.91, 1.39)	1.00	**1.16 (1.01**, 1.34)^*^	**1.41 (1.08**, 1.84)^*^
***Adjusted OR***^***b***^	***1.14 (0.90, 1.44)***	***1.00***	1.19 (1.02, 1.39)^*^	1.45 (1.08, 1.95)^*^
Adjusted OR ^c^	1.14 (0.90, 1.44)	1.00	1.17 (1.00, 1.37)^*^	**1.40 (1.04**, 1.89)^*^
Adjusted OR ^d^	1.08 (0.85, 1.37)	1.00	1.15 (0.98, 1.35)	**1.39 (1.02**, 1.88)^*^
Anemia at 12 months ^a^	85 (5.6)	549 (5.1)	235 (5.7)	35 (4.8)
Crude OR	1.12 (0.89, 1.42)	1.00	1.13 (0.97, 1.32)	0.94 (0.66, 1.33)
***Adjusted OR***^***b***^	***1.02 (0.78, 1.34)***	***1.00***	***1.13 (0.94, 1.34)***	***1.01 (0.70, 1.48)***
Adjusted OR ^c^	1.04 (0.80, 1.37)	1.00	1.12 (0.94, 1.33)	0.98 (0.67, 1.43)
Adjusted OR ^d^	1.02 (0.78, 1.34)	1.00	1.11 (0.93, 1.33)	0.99 (0.68, 1.45)

^a^ The proportions were calculated for infant anemia

^b^ Adjusted covariates were maternal age, ethnicity, education status, occupation, micronutrient supplementation, and infant’s sex, gestational age, feeding mode, and age of infant at hemoglobin measurement

^c^ Additionally adjusted for rate of gestational weight gain, mode of delivery, and birth weight

^d^ Further adjusted for mid-pregnancy anemia and gestational week when hemoglobin was measured during pregnancy

^*^P < 0.05

The analyses regarding association of maternal obesity with infant anemia at 6 months were repeated using BMI cutoffs for western populations recommended by the WHO [30], and the pattern was similar. However, a significantly increased risk of infant anemia at 6 months was observed for maternal overweight (adjusted OR 1.30, 95% CI 1.06, 1.60) rather than obesity (adjusted OR 1.39, 95% CI 0.87, 2.24), likely due to an inadequate sample size (*n* = 279) in the obese group (Additional file 2: Table S2). When women were reclassified ten subgroups (Q1 – Q10) with Q7 (22.2–22.9 kg/m^2^) as the reference group, results were not materially changed (Additional file 2: Table S3).

Maternal underweight seemed not to increase anemia risk for infants at 6 months (adjusted OR 1.08, 95% CI 0.85, 1.37) or 12 months (adjusted OR 1.02, 95% CI 0.78, 1.34) in this well-nourished population that did not include women with moderate or severe anemia (Table 3).

## Discussion

In this Chinese birth cohort, we found an inverse U-shaped relation between maternal BMI during early pregnancy and hemoglobin levels for infants at 6 months, persisting till 12 months. When hemoglobin levels were dichotomized, maternal obesity was associated with a 39% increased anemia risk for infants at 6 months but not 12 months. Analyses were repeated using BMI criteria for western population to define overweight and obesity, and results were not materially changed, indicating the robustness of the findings. Relevant results for 12-month old infant anemia were not statistically significant, probably due to weakening impacts of prenatal factors or manifesting impacts of postnatal factors (e.g. complementary foods or treatment of infant anemia at 6 months). We further found that maternal anemia during mid-pregnancy, though it linked to offspring anemia [29], aggravated the anemia risk for 6-month old infants, but did not mediated the association between maternal obesity and infant anemia at 6 months. Concerning maternal underweight, it seemed not to increase infant anemia risk in this relatively well-nourished population that excluded women with moderate or severe anemia.

Although no previous studies have assessed the relation of maternal BMI with offspring hemoglobin or anemia, some studies examined its relation with iron-related indices in cord blood [11–14]. Two prospective studies with a sample of < 316 mother-infant pairs from the United States showed a negative linear relation between maternal BMI at delivery and plasma ferritin in umbilical cord blood [11, 13]. A case-control study from the United States showed a negative relation between maternal BMI before pregnancy and iron status in cord blood, including serum iron and transferrin saturation [14]. The fourth study from China, a prospective analysis of 1613 mother-infant pairs, showed again a negative association of maternal BMI before pregnancy with cord blood ferritin levels [12]. These previous studies consistently showed that women with higher BMI were more likely to deliver neonates with lower iron stores at birth.

Additionally, the inverse U-shaped relation could also lend support from biological pathways. Low maternal BMI is often related to various micronutrient deficiencies including iron, vitamin A or B6 [31, 32]. These deficiencies interfere with iron mobilization and utilization in the process of hemoglobin synthesis to increase maternal anemia risk [33, 34] and then to increase offspring anemia risk [29]. On the other hand, high maternal BMI is regarded as a chronic inflammation status [35] which could lead to an increasing level of hepcidin, a crucial hormone regulating iron metabolism [36, 37]. The higher hepcidin level in blood might result in less iron absorption in intestine, less iron storage in hepatocytes, and less iron release from splenic macrophages [14], leading to low iron levels in mothers and consequently low iron stores in newborns [38]. In addition, women with high BMI are more likely to deliver a baby by cesarean [39] that affects placental transfusion or bacterial richness in newborns [40], leading to low hemoglobin levels in cord blood [41] or restricting dietary iron absorption and retention after birth [42].

Our study has some limitations. Firstly, this study was conducted within a randomized clinical trial of prenatal micronutrient supplementation that excluded women with a hemoglobin < 10.0 g/dl, likely restricting the generalization of our results to women with moderate or severe anemia during the first trimester of pregnancy. Secondly, two-thirds of the participants were provided with iron-containing supplementation, but the study design ensured that supplementation type was not associated with maternal BMI. Besides, we did not show a significant interaction between early pregnancy BMI and supplementation type (*P*_-interaction_ = 0.220; Additional file 2: Table S4). Thus, the associations were not likely biased by the provision of supplementation, but they do not necessarily apply to populations in which extensive supplementation is routine. Thirdly, the excluded women were more likely to have a higher BMI as compared to those included; if high BMI was associated with a higher risk of infant anemia, our estimate of the association between maternal BMI and infant anemia was likely underestimated. Fourthly, data on diet, physical activity and complications during pregnancy, as well as duration of breastfeeding were not recorded. In addition, the sample size in some subgroups might be insufficient, potentially affecting the robustness of the results in stratified analysis. Our study also has several strengths. To the best of our knowledge, this is the first study to investigate the relations of maternal BMI during early pregnancy with offspring hemoglobin levels and anemia risk. Infant hemoglobin and maternal key variables including maternal height, weight and hemoglobin were all measured by trained healthcare providers using unified equipment. Information on all incorporated covariates was from a surveillance system.

## Conclusions

In summary, the relation between maternal BMI during early pregnancy and infant hemoglobin is inversely U-shaped, with an apex at 22.4 (95% CI 21.6, 23.2) kg/m^2^ of maternal BMI in 6-month old infants. Maternal obesity was associated with an increased risk of infant anemia at 6 months, which seems to be aggravated by maternal anemia during pregnancy. Our study enriched the epidemiological evidence on the adverse effect of high maternal BMI on long-term health of offspring. More studies in other populations should be conducted to confirm or refute our findings.

## Supplementary information

**Additional file 1 Fig. S1**. Summary of mediation analysis. The a and S_a_ respectively represent logistic regression coefficients and corresponding standard errors in relation of maternal BMI to maternal anemia, and the b and S_b_ respectively represent logistic regression coefficients and corresponding standard errors in relation of maternal anemia to infant anemia at 6 months.

**Additional file 2 Table S1**. Adjusted ORs (95% CI) of infant anemia, stratified by maternal anemia during mid-pregnancy. **Table S2**. Crude and adjusted ORs (95% CI) of 6-month old infant anemia, using cutoffs for western population. **Table S3**. Crude and adjusted ORs (95% CI) of infant anemia for maternal BMI during early pregnancy categorized by deciles. **Table S4**. Adjusted ORs (95% CI) of 6-month old infant anemia, stratified by supplementation type.

## Data Availability

Data and material are available under request.

## Abbreviations

BMI: Body mass index
95% CI: 95% confidence interval
OR: odds ratio
GWG: gestational weight gain
WHO: World Health Organization
SD: standard deviation
IQR: interquartile range

## References

[1] Koyanagi A, Zhang J, Dagvadorj A, Hirayama F, Shibuya K, Souza JP, Gulmezoglu AM (2013). Macrosomia in 23 developing countries: an analysis of a multicountry, facility-based, cross-sectional survey. Lancet.

[2] He Y, Pan A, Yang Y, Wang YY, Xu JH, Zhang Y, Liu DJ, Wang QM, Shen HP, Zhang YP (2016). Prevalence of underweight, overweight, and obesity among reproductive-age women and adolescent girls in rural China. Am J Public Health.

[3] Lisonkova S, Muraca GM, Potts J, Liauw J, Chan WS, Skoll A, Lim KI (2017). Association between Prepregnancy body mass index and severe maternal morbidity. JAMA.

[4] Flenady V, Koopmans L, Middleton P, Froen JF, Smith GC, Gibbons K, Coory M, Gordon A, Ellwood D, McIntyre HD (2011). Major risk factors for stillbirth in high-income countries: a systematic review and meta-analysis. Lancet.

[5] Adane AA, Mishra GD, Tooth LR (2016). Maternal pre-pregnancy obesity and childhood physical and cognitive development of children: a systematic review. Int J Obes.

[6] Bodnar LM, Scanlon KS, Freedman DS, Siegariz AM, Cogswell ME (2001). High prevalence of postpartum anemia among low-income women in the United States. Am J Obstetr Gynecol.

[7] Bodnar LM, Siega-Riz AM, Miller WC, Cogswell ME, Mcdonald T (2002). Who should be screened for postpartum anemia? An evaluation of current recommendations. Am J Epidemiol.

[8] Garcia-Valdes L, Campoy C, Hayes H, Florido J, Rusanova I, Miranda MT, Mcardle HJ (2015). The impact of maternal obesity on iron status, placental transferrin receptor expression and hepcidin expression in human pregnancy. Int J Obes.

[9] Meinzen-Derr JK, Guerrero ML, Altaye M, Ortega-Gallegos H, Ruiz-Palacios GM, Morrow AL (2006). risk of infant anemia is associated with exclusive breast-feeding and maternal anemia in a Mexican cohort. J Nutr.

[10] Ntenda PAM, Nkoka O, Bass P, Senghore T (2018). Maternal anemia is a potential risk factor for anemia in children aged 6-59 months in southern Africa: a multilevel analysis. BMC Public Health.

[11] Dosch NC, Guslits EF, Weber MB, Murray SE, Ha B, Coe CL, Auger AP, Kling PJ (2016). Maternal obesity affects inflammatory and Iron indices in umbilical cord blood. J Pediatr.

[12] Jones AD, Zhao G, Jiang YP, Zhou M, Xu G, Kaciroti N, Zhang Z, Lozoff B (2016). Maternal obesity during pregnancy is negatively associated with maternal and neonatal iron status. Eur J Clin Nutr.

[13] Phillips AK, Roy SC, Lundberg R, Guilbert TW, Auger AP, Blohowiak SE, Coe CL, Kling PJ (2014). Neonatal iron status is impaired by maternal obesity and excessive weight gain during pregnancy. J Perinatol.

[14] Dao MC, Sen S, Iyer C, Klebenov D, Meydani SN (2013). Obesity during pregnancy and fetal Iron status: is Hepcidin the link?. J Perinatol.

[15] De Benoist B, Cogswell M, Egli I, Mclean E (2008). Worldwide prevalence of anaemia 1993-2005. WHO Glob Datab Anaemia.

[16] Kroker-Lobos MF, Pedroza-Tobias A, Pedraza LS, Rivera JA (2014). The double burden of undernutrition and excess body weight in Mexico. Am J Clin Nutr.

[17] Al-Mehaisen L, Khader Y, Al-Kuran O, Abu Issa F, Amarin Z (2011). Maternal anemia in rural Jordan: room for improvement. Anemia.

[18] Rezk M, Marawan H, Dawood R, Masood A, Abo-Elnasr M (2015). Prevalence and risk factors of iron-deficiency anaemia among pregnant women in rural districts of Menoufia governorate, Egypt. J Obstet Gynaecol.

[19] Stevens GA, Finucane MM, Deregil LM, Paciorek CJ, Flaxman SR, Branca F, Peñarosas JP, Bhutta ZA, Ezzati M (2013). Global, regional, and national trends in haemoglobin concentration and prevalence of total and severe anaemia in children and pregnant and non-pregnant women for 1995–2011: a systematic analysis of population-representative data. Lancet Global Health.

[20] Carter RC, Jacobson JL, Burden MJ, Rinat AS, Dodge NC, Mary Lu A, Betsy L, Jacobson SW (2010). Iron deficiency anemia and cognitive function in infancy. Pediatrics.

[21] Goobie SM, Faraoni D, Zurakowski D, DiNardo JA (2016). Association of preoperative Anemia with postoperative mortality in neonates. JAMA Pediatr.

[22] Liu JM, Mei ZG, Ye RW, Serdula MK, Ren AG, Cogswell ME (2013). Micronutrient supplementation and pregnancy outcomes: double-blind randomized controlled trial in China. JAMA Intern Med.

[23] Abrams B, Carmichael S, Selvin S (1995). Factors associated with the pattern of maternal weight gain during pregnancy. Obstet Gynecol.

[24] Tan K (2004). Consultation we: appropriate body-mass index for Asian populations and its implications for policy and intervention strategies. Lancet.

[25] World Health Organization: Haemoglobin concentrations for the diagnosis of anaemia and assessment of severity. 2011. www.who.int/vmnis/indicators/haemoglobin/en/index.html.

[26] Shewhart WA, Wilks SS: Multivariable Model-Building: A pragmatic approach to regression analysis based on fractional polynomials for modelling continuous variables. 2008.

[27] Royston P, Ambler G, Sauerbrei W (1999). The use of fractional polynomials to model continuous risk variables in epidemiology. Int J Epidemiol.

[28] World Health Organization: Iron deficiency anaemia: assessment prevention and control. A guide for programme managers. 2001. https://www.who.int/nutrition/publications/micronutrients/anaemia_iron_deficiency/WHO_NHD_01.3/en/.

[29] Saskia DP, Bloem MW, Mayang S, Lynnda K, Ray Y, Soewarta K (2002). The high prevalence of low hemoglobin concentration among Indonesian infants aged 3-5 months is related to maternal anemia. J Nutr.

[30] World Health Organization: Obesity: preventing and managing the global epidemic. Report of a WHO consultation. Geneva World Health Organ 1998, 15:18–30. 11234459

[31] Huddle JM, Gibson RS, Cullinan TR (1999). The impact of malarial infection and diet on the anaemia status of rural pregnant Malawian women. Eur J Clin Nutr.

[32] Gibson RS, Abebe Y, Stabler S, Allen RH, Westcott JE, Stoecker BJ, Krebs NF, Hambidge KM (2008). Zinc, Gravida, infection, and Iron, but not vitamin B-12 or Folate status, predict hemoglobin during pregnancy in southern Ethiopia. J Nutr.

[33] Suharno D, West CE, Muhilal Karyadi D, JGAJ H (1993). supplementation with vitamin-a and Iron for nutritional Anemia in pregnant-women in West-Java, Indonesia. Lancet.

[34] Hisano M, Suzuki R, Sago H, Murashima A, Yamaguchi K (2010). Vitamin B6 deficiency and anemia in pregnancy. Eur J Clin Nutr.

[35] Hotamisligil GS, Shargill NS, Spiegelman BM (1993). Adipose expression of tumor-necrosis-factor-alpha - direct role in obesity-linked insulin resistance. Science.

[36] Bekri S, Gual P, Anty R, Luciani N, Dahman M, Ramesh B, Iannelli A, Staccini-Myx A, Casanova D, Ben Amor I (2006). Increased adipose tissue expression of hepcidin in severe obesity is independent from diabetes and NASH. Gastroenterology.

[37] Laine F, Deugnier Y (2006). Increased expression of hepcidin in obese patients: impact on phenotypic expression of hemochromatosis and pathophysiology of dysmetabolic iron overload syndrome. Gastroenterology.

[38] Shao J, Lou JA, Rao R, Georgieff MK, Kaciroti N, Felt BT, Zhao ZY, Lozoff B (2012). Maternal serum ferritin concentration is positively associated with newborn Iron Stores in Women with low ferritin status in late pregnancy. J Nutr.

[39] Zhou YB, Blustein J, Li HT, Ye RW, Zhu LP, Liu JM (2015). Maternal obesity, caesarean delivery and caesarean delivery on maternal request: a cohort analysis from China. Paediatr Perinat Epidemiol.

[40] Azad MB, Konya T, Maughan H, Guttman DS, Field CJ, Chari RS, Sears MR, Becker AB, Scott JA, Kozyrskyj AL, Investigators CS (2013). Gut microbiota of healthy Canadian infants: profiles by mode of delivery and infant diet at 4 months. Can Med Assoc J.

[41] Zhou YB, Li HT, Zhu LP, Liu JM (2014). Impact of cesarean section on placental transfusion and iron-related hematological indices in term neonates: a systematic review and meta-analysis. Placenta.

[42] Reddy BS, Pleasants JR, Wostmann BS (1972). Effect of intestinal microflora on iron and zinc metabolism, and on activities of metalloenzymes in rats. J Nutr.

