# Supplementary material for: Association of maternal BMI during early pregnancy with infant anemia: a large Chinese birth cohort
Source: Nutr Metab (Lond). 2020 Apr 19;17:32. doi: 10.1186/s12986-020-00448-w (PMC7169019; doi:10.1186/s12986-020-00448-w)
Supplement: Supplementary file 2 — Additional file 2 Table S1. Adjusted ORs (95% CI) of infant anemia, stratified by maternal anemia during mid-pregnancy. Table S2. Crude and adjusted ORs (95% CI) of 6-month old infant anemia, using cutoffs for western population. Table S3. Crude and adjusted ORs (95% CI) of infant anemia for maternal BMI during early pregnancy categorized by deciles. Table S4. Adjusted ORs (95% CI) of 6-month old infant anemia, stratified by supplementation type. [file 12986_2020_448_MOESM2_ESM.doc]

**Table S1**. Adjusted ORs (95% CI) of 6-month old infant anemia, stratified by maternal anemia or not during mid-pregnancy

|  | Total No. | No. of case (%) | Adjusted OR ^a^ | *P*_-interaction_ |
| --- | --- | --- | --- | --- |
| Women with anemia in mid-pregnancy |  |  |  |  |
| Underweight | 119 | 17 (14.3) | 0.74 (0.38, 1.48) | 0.130 |
| Normal weight | 670 | 95 (14.2) | 1.00 |
| Overweight | 256 | 30 (11.7) | 0.90 (0.55, 1.47) |
| Obesity | 37 | 9 (24.3) | **2.91 (1.14**, **7.46)^*^** |
| Women without anemia in mid-pregnancy |  |  |  |
| Underweight | 1388 | 90 (6.5) | 1.13 (0.88, 1.46) |
| Normal weight | 10 159 | 593 (5.8) | 1.00 |
| Overweight | 3868 | 271 (7.0) | **1.19 (1.01, 1.41)*** |
| Obesity | 696 | 55 (7.9) | 1.28 (0.92, 1.77) |

^a^ Adjusted covariates were maternal age, ethnicity, education status, occupation, micronutrient supplementation, rate of gestational weight gain, gestational week when hemoglobin was measured during pregnancy, and infants’ sex, gestational age, mode of delivery, birth weight, feeding mode, and age of infant at hemoglobin measurement.

^*^ *P* < 0.05

**Table S2**. Crude and adjusted ORs (95% CI) of 6-month old infant anemia, using cutoffs for western population a

| BMI during early pregnancy | Total No. | No. of case (%) | Crude OR | Adjusted OR ^b^ | Adjusted OR ^c^ |
| --- | --- | --- | --- | --- | --- |
| Underweight | 1507 | 107 (7.1) | 1.11 (0.90, 1.37) | 1.11 (0.88, 1.41) | 1.06 (0.84, 1.34) |
| Normal weight | 13 699 | 881 (6.4) | 1.00 | 1.00 | 1.00 |
| Overweight | 1708 | 149 (8.7) | **1.39 (1.16**, **1.67)^*^** | **1.35 (1.10**, **1.66)^*^** | **1.30 (1.06**, **1.60)^*^** |
| Obesity | 279 | 23 (8.3) | 1.31 (0.85, 2.03) | 1.36 (0.85, 2.18) | 1.39 (0.87, 2.24) |

^a^ The BMI was categorized as underweight (< 18.5 kg/m^2^), normal weight (18.5 – 24.9 kg/m^2^), overweight (25.0 – 29.9 kg/m^2^), and obesity (≥ 30.0 kg/m^2^). The proportions were calculated for infant anemia.

^b^ Adjusted covariates were maternal age, ethnicity, education status, occupation, micronutrient supplementation, rate of gestational weight gain, and infants’ sex, gestational age, mode of delivery, birth weight, feeding mode, and age of infant at hemoglobin measurement.

^c^ Additionally adjusted for mid-pregnancy anemia and gestational week when hemoglobin was measured during pregnancy.

^*^ *P* < 0.05

**Table S3**. Crude and adjusted ORs (95% CI) of infant anemia at 6 months for maternal BMI during early pregnancy categorized by deciles a

|  | Q1 | Q2 | Q3 | Q4 | Q5 | Q6 | Q7 | Q8 | Q9 | Q10 |
| --- | --- | --- | --- | --- | --- | --- | --- | --- | --- | --- |
| Anemia at 6 months ^b^ | 117 (6.7) | 126 (7.5) | 127 (7.3) | 93 (5.7) | 107 (6.1) | 118 (6.4) | 92 (5.3) | 95 (5.3) | 133 (7.8) | 150 (8.9) |
| Crude OR | 1.29  (0.97, 1.71) | **1.44**  **(1.09, 1.90)^*^** | **1.41**  **(1.07, 1.86) ^*^** | 1.09  (0.80, 1.46) | 1.16  (0.87, 1.54) | 1.22  (0.92, 1.62) | 1.00 | 1.06  (0.79, 1.43) | **1.51**  **(1.15, 1.98)** | **1.74**  **(1.33, 2.28)^*^** |
| Adjusted OR ^c^ | 1.33  (0.97, 1.83) | **1.47**  **(1.08, 2.01)^*^** | 1.35  (0.98, 1.85) | 1.17  (0.84, 1.63) | 1.13  (0.81, 1.56) | 1.28  (0.93, 1.75) | 1.00 | 1.12  (0.81, 0.56) | **1.57**  **(1.15, 2.13) ^*^** | **1.72**  **(1.27**, **2.34)^*^** |
| Adjusted OR ^d^ | 1.29  (0.94, 1.77) | **1.46**  **(1.06, 2.00)^*^** | 1.36  (0.99, 1.87) | 1.19  (0.85, 1.66) | 1.15  (0.83, 1.60) | 1.30  (0.95, 1.78) | 1.00 | 1.11  (0.80, 1.54) | **1.57**  **(1.15, 2.14) ^*^** | **1.70**  **(1.25, 2.31)^*^** |

^a^ Maternal BMI during early pregnancy was categorized into deciles: Q1 (< 18.7 kg/m^2^), Q2 (18.7 – 19.6 kg/m^2^), Q3 (19.7 – 20.3 kg/m^2^), Q4 (20.4 – 20.9 kg/m^2^), Q5 (21.0 – 21.5 kg/m^2^), Q6 (21.6 – 22.2 kg/m^2^), Q7 (22.3 – 22.9 kg/m^2^), Q8 (23.0 – 23.8 kg/m^2^), Q9 (23.9 – 25.4 kg/m^2^), Q10 (≥ 25.5 kg/m^2^).

^b^ The proportions were calculated for infant anemia.

^c^ Adjusted covariates were maternal age, ethnicity, education status, occupation, micronutrient supplementation, rate of gestational weight gain, and infant’s sex, gestational age, mode of delivery, birth weight, feeding mode, and age of infant at hemoglobin measurement.

^d^ Additionally adjusted for mid-pregnancy anemia and gestational week when hemoglobin was measured during pregnancy.

^*^ *P* < 0.05

**Table S4**. Adjusted ORs (95% CI) of 6-month old infant anemia, stratified by supplementation type

|  | Total No. | No. of case (%) | Adjusted OR ^a^ | *P*_-interaction_ |
| --- | --- | --- | --- | --- |
| Stratum with folic acid |  |  |  | 0.220 |
| Underweight | 515 | 47 (9.1) | 1.43 (0.98, 2.09) |
| Normal weight | 3610 | 231 (6.4) | 1.00 |
| Overweight | 1385 | 97 (7.0) | 1.07 (0.81, 1.42) |
| Obesity | 227 | 19 (8.4) | 1.39 (0.81, 2.38) |
| Stratum with iron-folic acid |  |  |  |
| Underweight | 505 | 30 (5.9) | 0.85 (0.53, 1.36) |
| Normal weight | 3613 | 224 (6.2) | 1.00 |
| Overweight | 1388 | 114 (8.2) | **1.41 (1.08, 1.84)*** |
| Obesity | 244 | 16 (6.6) | 1.18 (0.66, 2.09) |
| Stratum with multiple micronutrients |  |  |  |
| Underweight | 487 | 30 (6.2) | 0.95 (0.62, 1.07) |
| Normal weight | 3606 | 233 (6.5) | 1.00 |
| Overweight | 1351 | 90 (6.7) | 0.98 (0.74, 1.30) |
| Obesity | 262 | 29 (11.1) | 1.52 (0.93, 2.48) |

^a^ Adjusted covariates were maternal age, ethnicity, education status, occupation, rate of gestational weight gain, mid-pregnancy anemia, gestational week when hemoglobin was measured during pregnancy, and infants’ sex, gestational age, mode of delivery, birth weight, feeding mode, and age of infant at hemoglobin measurement.

^*^ *P* < 0.05
